# Dynamics and Potential Significance of Spontaneous Activity in the Habenula

**DOI:** 10.1523/ENEURO.0287-21.2022

**Published:** 2022-09-01

**Authors:** Suryadi, Ruey-Kuang Cheng, Elliot Birkett, Suresh Jesuthasan, Lock Yue Chew

**Affiliations:** 1 School of Physical and Mathematical Sciences, Nanyang Technological University, Singapore 637371; 2 Lee Kong Chian School of Medicine, Nanyang Technological University, Singapore 636921; 3 Institute of Molecular and Cell Biology, Singapore 138673; 4 School of Biosciences, University of Sheffield, Sheffield S10 2TN, United Kingdom; 5 Complexity Institute, Nanyang Technological University, Singapore 637335

**Keywords:** avalanche, criticality, dynamics, habenula, reverberation, spontaneous activity

## Abstract

The habenula is an evolutionarily conserved structure of the vertebrate brain that is essential for behavioral flexibility and mood control. It is spontaneously active and is able to access diverse states when the animal is exposed to sensory stimuli. Here we investigate the dynamics of habenula spontaneous activity, to gain insight into how sensitivity is optimized. Two-photon calcium imaging was performed in resting zebrafish larvae at single-cell resolution. An analysis of avalanches of inferred spikes suggests that the habenula is subcritical. Activity had low covariance and a small mean, arguing against dynamic criticality. A multiple regression estimator of autocorrelation time suggests that the habenula is neither fully asynchronous nor perfectly critical, but is reverberating. This pattern of dynamics may enable integration of information and high flexibility in the tuning of network properties, thus providing a potential mechanism for the optimal responses to a changing environment.

## Significance Statement

Spontaneous activity in neurons shapes the response to stimuli. One structure with a high level of spontaneous neuronal activity is the habenula, a regulator of broadly acting neuromodulators involved in mood and learning. How does this activity influence habenula function? We show here that the habenula of a resting animal is near criticality, in a state termed “reverberation”. This pattern of dynamics is consistent with high sensitivity and flexibility, and may enable the habenula to respond optimally to a wide range of stimuli.

## Introduction

A defining feature of neural circuits is their flexibility. Although anatomic connectivity does not change rapidly in a mature animal, functional connectivity can change within seconds, enabling animals to quickly alter their behavior to deal with changing circumstances. This is made possible in part by the action of neuromodulators (Getting, 1989; Marder, 2012; Bargmann and Marder, 2013). To ensure that functional connectivity is appropriate for a given condition, the release of neuromodulators is tied to a variety of factors, such as the internal state of the animal, external cues, and perceived reward.

The habenula is an epithalamic structure that receives indirect input from several sensory systems (Stephenson-Jones et al., 2012; Cheng et al., 2017; Zhang et al., 2017), is stimulated by punishment or absence of an expected reward (Matsumoto and Hikosaka, 2007; Hong and Hikosaka, 2008; Amo et al., 2014), and has activity that is dependent on the circadian clock (Zhao and Rusak, 2005; Baño-Otálora and Piggins, 2017; Basnakova et al., 2021) and stress levels (Authement et al., 2018). It sends output to a number of structures, including the raphe (Wang and Aghajanian, 1977) and the rostromedial tegmental nucleus (Jhou et al., 2009), which regulate the release of serotonin and dopamine, respectively. Thus, the habenula is well positioned to coordinate release of broadly acting neuromodulators based on a variety of factors. The importance of the habenula is illustrated by a range of defects seen in animals with a lesioned habenula, including an inability to cope with changing circumstances (Thornton and Bradbury, 1989) or to learn effectively (Agetsuma et al., 2010; Lee et al., 2010). The habenula is thus required for behavioral flexibility (Palumbo et al., 2020; Hones and Mizumori, 2022).

How does the habenula process information to efficiently influence behavior? Calcium imaging in zebrafish indicates that neural activity in the habenula is sensitive to various features of sensory input. Different concentrations of odors (Krishnan et al., 2014), as well as wavelengths (Lin, 2015) or levels of ambient illumination (Zhang et al., 2017) trigger different patterns of activity. This can be represented as trajectories in state space, with reproducible trajectories for each stimulus, indicating that sensory stimuli are represented by population activity. Additionally, habenula neurons constantly fire action potentials, even in the absence of sensory stimulation. This was demonstrated in rats using electrical recordings of brain slices, and has also been found in the zebrafish, using two-photon calcium imaging of tethered animals (Jetti et al., 2014; Fore et al., 2018), or CaMPARI (Calcium Modulated Photoactivatable Ratiometric Integrator)-based labeling of freely swimming fish (Fosque et al., 2015). This activity is driven by input from the forebrain (Bartoszek et al., 2021).

The dynamics of spontaneous activity can influence how information is processed. For example, it has been suggested that highly decorrelated firing, also termed “asynchronous irregular activity,” allows a network to respond rapidly to new input, as the effects of previous inputs are quickly dissipated (Van Vreeswijk and Sompolinsky, 1996). Alternatively, the network may reside close to a point of phase transition, as proposed by criticality theory (Beggs and Timme, 2012). However, whether such a state exists *in vivo* is controversial (Wilting and Priesemann, 2019b). A third possibility is that the network resides at a near critical yet subcritical point between these two regimes, termed reverberation (Wilting and Priesemann, 2018).

To determine the dynamics of the resting habenula, we assess activity using calcium imaging in the zebrafish. The transparency of this system allows imaging in the intact animal. To mitigate inaccuracies that arise from subsampling (Priesemann et al., 2009; Ribeiro et al., 2014), which can lead to failure to detect criticality, we use the MR estimator that was recently described (Wilting and Priesemann, 2018).

## Materials and Methods

### Ethical statement

Experiments were conducted using protocols approved by the Institutional Animal Care and Use Committee of Biopolis, Singapore (#171215).

### Microscopy and image processing

Calcium imaging was conducted on an upright two-photon microscope system (model A1RMP, Nikon) with a 25× water-immersion objective. Fish expressing nuclear-localized GCaMP6s under the control of the *elavl3* promoter [i.e., *Tg(elavl3:H2B-GCaMP6s)*; Dunn et al., 2016; age, 7–11 d] were embedded dorsal up in 2% agarose. At this age, sex cannot be determined (Santos et al., 2017). Time-lapse images were collected with no delay between frames, using a resonant scanner. A single plane was recorded at 113 Hz (*n* = 2) or ∼15 Hz (*n* = 16) for 10–15 min. The *x–y* spatial resolution was 0.50 μm/pixel for 113 Hz data and 0.33 μm/pixel for ∼15 Hz data.

All processing steps for the recordings, including motion correction and detection of regions of interest (ROIs), were done using suite2p (Pachitariu et al., 2017) with the ROIs subsequently manually curated to exclude neurons outside the region of the habenula. From the fluorescence signals obtained via suite2p, the corresponding Δ*F*/*F* traces were then computed (Jia et al., 2011), following which discrete spikes were inferred using MLspike with gCaMP6s parameters given in the article (Deneux et al., 2016). Over all of the recordings, the number of neurons range from 152 to 252 with a mean value of 205.6.

### Avalanche analysis

Neuronal avalanches were computed by summing the neural spike activities across the population, with individual avalanches being separated by periods of silent time bins (i.e., with zero activity). The total number of spikes across one avalanche constitutes the size, while the duration measures the number of time points the avalanche lasts. The power law distribution was fitted and compared against the exponential distribution using the powerlaw package (Alstott et al., 2014). As the mean size given the duration is not a probability distribution, the function was fitted with a simple linear regression in the log scale.

Shape collapse was performed by adapting the procedure described in the study by Marshall et al. (2016) into Python. We began by excluding avalanches whose durations are less than five timesteps, as well as durations whose total number of avalanche samples are less than three. This was followed by computing the average avalanche profile for all durations, after which the durations were rescaled to one. Next, linear interpolation was performed for each avalanche profile at 1000 points across the rescaled duration. This allows the computation of the variance across avalanche profiles through the interpolated points. The shape collapse error could then be computed, defined as the ratio between the mean variance and the squared span of the avalanche profiles. Here the span refers to the difference between the maximum and minimum values over all the avalanche profiles. Using this shape collapse error, we then searched for the parameter *γ* that minimizes the shape collapse error in the interval 0 ≤ *γ* ≤ 5 in increasing precision: first in steps of 0.1, then 0.01, and last 0.001.

### Branching parameter estimation

A branching process is defined as a process where the number of active units in the next timestep *A_t_*_+1_ (i.e., descendants) is on average a constant multiple of the active units in the current timestep *A_t_* (i.e., ancestors). We denote this constant *m*, which is also called the branching parameter. Mathematically we represent it as follows:

(1) $$\langle A_{t+1}|A_{t}\rangle =mA_{t}+h,$$

where *h* represents the mean rate of an external drive coming from outside the branching dynamics, which in the brain may come from stimuli or other brain regions. As the equation suggests, the branching parameter *m* determines the stability of the dynamics, ranging from one with exponential decay (*m *<* *1) to exponential growth (*m *>* *1). *m *=* *1 then denotes the critical boundary between the two dynamics, which is where critical avalanches in neuronal systems are said to lie in the study by Beggs and Plenz (2003).

Under spatial subsampling, the conventional way of simply regressing the time series at one timestep apart (i.e., *A_t_*_+1_ against *A_t_*) fails to accurately determine *m*. Here we make use of the multistep regression (MR) estimator of *m* that has been proven to be consistent even under severe binomial subsampling (Wilting and Priesemann, 2018). To do so, linear regressions were performed for all pairs of *A_t_*_+_*_k_* against *A_t_* for all *k *=* *1, …, *k*_max_ to obtain slopes *r_k_*. It has been shown that while under full sampling we have $r_{k}=m^{k}$, subsampling leads to a constant bias for the slope *r_k_* at all values of *k* (Wilting and Priesemann, 2018), as follows:

(2) $$r_{k}=bm^{k}.$$

The true *m* can then be readily estimated by curve fitting *r_k_* against *k* using this equation. We note that since 0 ≤ *m *≤* *1, this relation is in general an exponential decay function, except when *m *=* *0 or 1. *m^k^* can also be rewritten as *e*^–^ *^k^*^Δ^*^t^*^/^*^τ^* (Wilting and Priesemann, 2018), where *τ* = – Δ*t*/log *m* denotes the autocorrelation time of the process and Δ*t* is the timescale of the data (i.e., the time interval between each measured timestep). *τ* diverges at criticality (when *m *=* *1), and otherwise still yields information about the timescale at which the influence of a perturbation remains in the system.

### Tests on nonstationarity and Poisson activity

As with most statistical estimations for time series, this estimator is valid only when the time series is stationary. We applied a series of tests to identify common sources of nonstationarities (tests 1–3) and to check for the possible presence of Poisson activity (test 4; Wilting and Priesemann, 2018). Our aim was to ensure that the branching process is able to describe the dynamics present in the data. The following was done. (1) We tested the original exponential model $r_{k}=b\cdot m^{k}$ against the exponential model $r_{k}=b_{\text{offset}}\cdot m_{\text{offset}}^{k}+c_{\text{offset}}$, with offset *c*_offset_ accounting for a transient increase in the drive. If the residual of the fit with offset $R_{\text{offset}}^{2}$ was smaller than the residual of the original model $R_{\text{exp}}^{2}$ by a factor of two, $H_{\text{offset}}=(2\cdot R_{\text{offset}}^{2}<R_{\text{exp}}^{2})$, we rejected the dataset. (2) We compared the relative difference between the autocorrelation time *τ* of the original exponential model and the above exponential model with offset to detect ramping of the drive. If the difference was too large, $H_{\tau }=(|\tau_{\text{exp}}-\tau_{\text{offset}}|/\min\{\tau_{\text{exp}},\tau_{\text{offset}}\}>2)$, we again deduced the invalidity of the MR estimation. (3) If the linear regression model $r_{k}=q_{1}k+q_{2}$ gave a better fit of *r_k_* against *k* compared with the original exponential model such that the residuals $R_{\text{lin}}^{2}$ was smaller than $R_{\text{exp}}^{2}$: $H_{\text{lin}}=(R_{\text{lin}}^{2}<R_{\text{exp}}^{2})$, we rejected the data and concluded that the presence of sudden jumps in the drive were due to state changes. (4) We performed a one-sided *t* test to determine whether the mean of *r_k_* is significantly larger than zero, which is useful when *r_k_* appears as a flat line as it may result from both critical (*m* = 1, *r_k_* > 0) and Poisson (*m* = 0, *r_k_* = 0) cases. The *t* test returns a *p*-value $p_{\bar{r}\leq 0}$, through which we define a test *H*_MR_invalid_ $=(p_{\bar{r}\leq 0}\geq 0.1)$. If *H*_MR_invalid_ is positive, Poisson activity is then confirmed by performing an additional test to ensure that no systematic trend exists in *r_k_*. This is done via the above linear regression model by showing that *r_k_* has slope *q*_1_ zero with $H_{\text{poisson}}=(p_{q_{1}=0}\geq 0.05)$. Datasets that are positive for both *H*_MR_invalid_ and *H*_poisson_ are explained by Poisson activity, while those positive for *H*_MR_invalid_ but not *H*_poisson_ are not Poisson but are nevertheless invalid for MR estimation.

Nonstationarities are uncovered when any of the computed *H*_offset_, *H_τ_*, or *H*_lin_ values is positive. MR estimation is considered valid when *H*_offset_, *H_τ_*, *H*_lin_, and *H*_MR_invalid_ values are all negative (regardless of *H*_poisson_). When both *H*_MR_invalid_ and *H*_poisson_ values are positive, the procedure is valid with the conclusion that the system exhibits Poisson activity (i.e., *m* = 0). On the other hand, if *H*_MR_invalid_ is positive but *H*_poisson_ is negative, then MR estimation is not valid and no further conclusion can be made on the activity.

### Spike count covariance

The spike count covariance was computed as described in the study by Dahmen et al. (2019). First, each dataset was divided into *N* subsets, and the total activity was computed for each neuron in each subset, which we denoted as $n_{i}^{p}$, where *i* denotes the neuron and *p *=* *1, …, *N* denotes the subset. We then computed for each combination of subsets the unbiased estimator for the covariance between neuron *i* and neuron *j*, as follows:

(3) $${\hat{c}}_{ij}=\frac{1}{N-1}\sum_{p=1}^{N}\left(n_{i}^{p}-{\bar{n}}_{i}\right)\;(n_{j}^{p}-{\bar{n}}_{j}),$$

where ${\bar{n}}_{i}=\frac{1}{N}\sum_{p=1}^{N}n_{i}^{p}$ denotes the average total activity of neuron *i* over the different subsets. The corresponding spike count correlation could be computed by dividing each ${\bar{n}}_{i}$ by the square root of its variance $\sqrt{{\hat{c}}_{ii}}$. The dimensionality *N*_eff_ of the activity could be measured via the participation ratio (Abbott et al., 2011; Mazzucato et al., 2016). This is determined by performing principal component analysis (PCA) on the spike count matrix, and calculating the following:

(4) $$N_{\text{eff}}={\left(\sum_{k=1}^{K}{\tilde{\lambda }}_{k}^{2}\right)}^{-1},$$

with ${\tilde{\lambda }}_{k}$ being the explained variance ratio of the *k*th principal component.

We can probe how far the system is to the dynamically balanced critical state through *λ*_max_, which measures the largest (and therefore most unstable) eigenvalue of the underlying connectivity matrix of the network. The critical point occurs at *λ*_max_ = 1. It is given by Dahmen et al. (2019), as follows:

(5) $$\lambda_{\max}=\sqrt{1-\sqrt{\frac{1}{1+N\Delta^{2}}}},$$

where $\Delta =\delta c_{ij}/c_{ii}$ is the normalized width of the spike count covariance distribution with *δc_ij_* being the SD of the covariance distribution, and *N* is the underlying network size, which we take to be 1500 for the larval zebrafish habenula (Pandey et al., 2018). A limited amount of data incurs bias in the estimation of the width; an extensive discussion on the bias and its correction can be found in the study by Dahmen et al. (2019).

### Data availability

Multistep regression was performed using the MR estimator toolbox (Spitzner et al., 2021). The remaining codes used for computation in this study are available at GitHub (https://github.com/suryadi-t/reverberating_habenula). All computations were performed on an HP Z440 Workstation (3.70 GHz CPU) with the 64 bit Windows 10 Education OS.

## Results

### Analysis of avalanches fails to provide evidence of criticality

**Figure 1.**
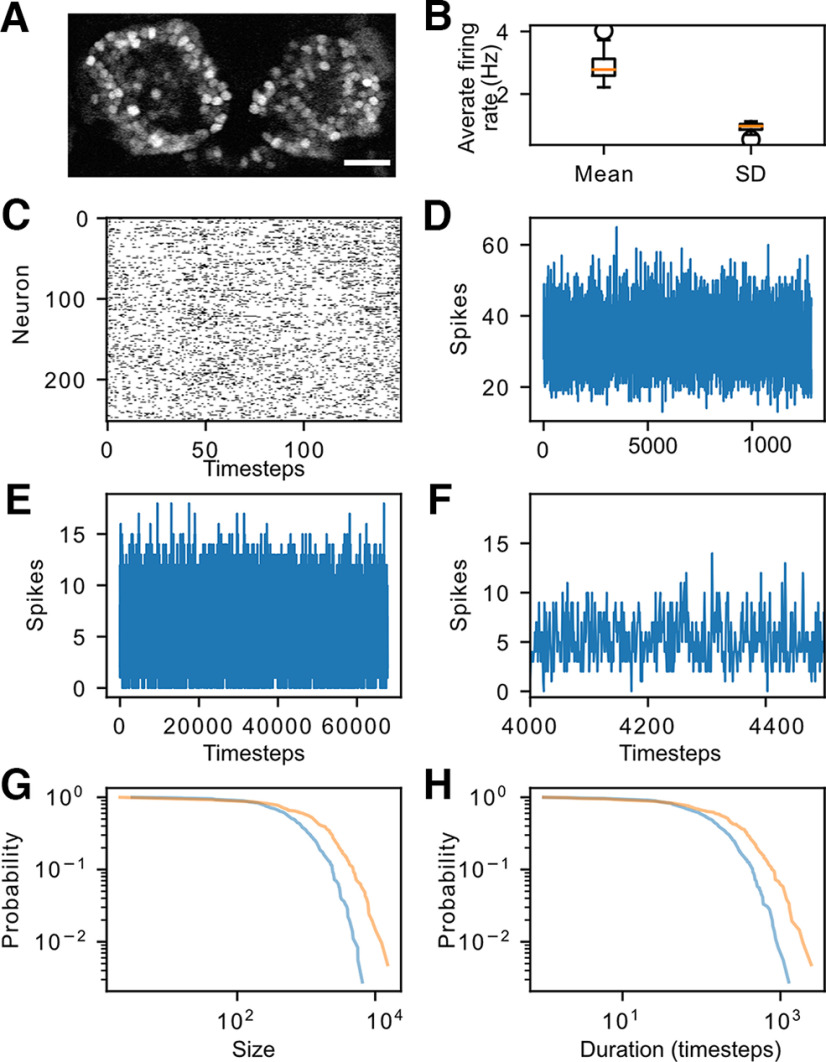
Characterization of neuronal avalanches in the habenula. ***A***, The habenula of *Tg(elavl3:H2B-GCaMP6s)* fish, imaged at 15 Hz. Scale bar, 25 μm. ***B***, Population statistics (mean and standard deviation, SD) of the neuron average firing rate aggregated over all recordings. The individual firing rate distributions for each recording are given in Extended Data Figure 1-1. ***C***, Activity in a subset of neurons in the habenula imaged at 15 Hz. The black lines indicate frames where there is at least one spike inferred by MLspike. ***D***, Plot showing number of spikes across the population as inferred by MLspike. ***E***, ***F***, Inferred spikes, obtained from imaging a single plane at 113 Hz. ***E*** is the entire recording, whereas ***F*** is a zoomed in version to a portion. ***G***, ***H***, Distribution of avalanche size (***G***) and duration (***H***) in fish imaged at 113 Hz at a single plane. The blue curve is based on data from one fish, while the orange curve is derived from a second fish. The log-log plots are not linear, indicating the absence of a power law. Further avalanche analyses are given in Extended Data Figure 1-2.

We first asked whether there is evidence for criticality in the habenula. One signature of criticality is the presence of neuronal avalanches, which is defined as cascades of neuronal activity between periods of silence, with duration and size distributions exhibiting power laws. Two-photon calcium imaging was used to monitor spontaneous activity in the zebrafish habenula (Fig. 1*A*), as visible light used in confocal microscopy causes an evoked response (Dreosti et al., 2014). Upon recording a single plane of neurons in the habenula (at ∼15 Hz) and using MLspike to infer spiking activity in the neurons, we found that at least one neuron was active at any given frame for all 16 recordings (Fig. 1*C*,*D*). This lack of silent time bins, which implies a lack of avalanches, could be because of the slow rate of recording. We thus made additional recordings at 113 Hz. These yielded periods of inactivity (Fig. 1*E*,*F*): two 10 min recordings yielded 360 and 207 distinct avalanches. These observations suggest that avalanches exist in the habenula.

10.1523/ENEURO.0287-21.2022.f1-1 Figure 1-1 Average firing rate distributions for each recording computed from the discrete spikes inferred using MLspike. Download Figure 1-1, TIF file.

**Figure 2.**
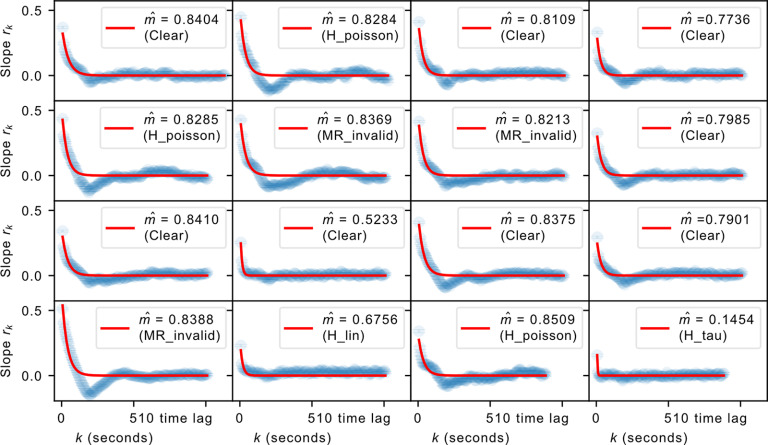
The regression slopes *r_k_* at different time lags *k*. The blue curve represents actual data, whereas the red curve is the fit using the estimated $\hat{m}$ based on Equation 2. The value of the estimated $\hat{m}$ is shown alongside the results for stationarity tests—datasets that pass all the tests are denoted as Clear while those that fail at least one are indicated by the first test they failed. For relatively short datasets, even a stationary branching process can test positive for *H*_poisson_ and *H*_MR_invalid_, as shown in Extended Data Figure 2-1.

10.1523/ENEURO.0287-21.2022.f1-2 Figure 1-2 Further avalanche analysis. Top row, Mean avalanche size given duration. Bottom row, Avalanche shape collapse. We see that *β* and *γ* are far from satisfying Equation 10, further indicating that the habenula is not critical. Download Figure 1-2, file.

It is predicted from scaling theory (and verified experimentally) that critical avalanching systems manifest power law distributions for both the avalanche size (*S*) and duration (*T*; Beggs and Plenz, 2003). In addition, the mean avalanche size given duration 〈S〉(T) also follows a power law (Friedman et al., 2012). These are summarized below:

(6) $$f(S)∼S^{-\tau },$$

(7) $$f(T)∼T^{-\alpha },$$

(8) $$〈S〉(T)∼T^{\beta }.$$

When analyzed with the powerlaw package (Alstott et al., 2014), the size and duration of these avalanches did not fit a power law (Fig. 1*G*,*H*; likelihood ratio test did not favor power law over exponential distribution for all cases with *p *>* *0.1). While we observe that Equation 8 appears to hold in this system (Extended Data Fig. 1-2), this relation is known to be valid quite far from criticality (Friedman et al., 2012), and is therefore insufficient on its own to demonstrate criticality. This can be analyzed further by inspecting the avalanche shape (Extended Data Fig. 1-2). A critical system exhibits self-similarity, which manifests not only power law avalanche distributions, but also avalanche shapes that are, on average, identical across scales. Mathematically, the mean number of spiking neurons *s*(*t*, *T*) at the *t*th time instant in an avalanche of duration *T* relates to a universal profile *F*(*t*/*T*) as follows:

(9) $$s(t,T)\propto T^{\gamma }F(t/T).$$

By rescaling the durations, these avalanches of different durations could then be collapsed into a universal shape by computing *s*(*t*, *T*)*T*^–^*^γ^*. In a critical avalanching system, the resulting *γ* relates to *β* from Equation 8 in a simple way (Friedman et al., 2012):

(10) γ=β−1.

We observe in our data that while there is an apparent shape collapse, the shape is flat instead of the parabolic shape predicted by mean field theory, which has also been observed experimentally in neural systems (Friedman et al., 2012; Marshall et al., 2016). In addition, Equation 10 fails to hold here. These observations, particularly the absence of power law distributions in both avalanche size and duration, indicate that the system is not critical.

### The MR estimator suggests a subcritical, reverberating state

This lack of evidence for a critical state may be because of subsampling, which was imposed by technical limitations. Specifically, volumetric imaging could not be conducted at a high enough rate, because of delays in focusing across the entire depth of the habenula. We thus used the MR estimator to provide a measure of where the dynamics of the habenula resides in the asynchronous versus critical spectrum. Given that nonstationarities in the data may lead to inaccuracies in the estimation process (Wilting and Priesemann, 2018), we first performed several tests to identify common nonstationarities. These results are indicated in Figure 2, where datasets that passed all stationarity tests are indicated as “Clear” while datasets that did not pass are indicated by the first test they failed. Here the points are expected to follow an exponential decay as given in Equation 2 in the Materials and Methods section, which we observe to be the case, with the red curve in each subplot representing the fit by the exponential decay function. We later show in Extended Data Figure 3-1 that this shape is abolished on temporally shuffling the data.

10.1523/ENEURO.0287-21.2022.f2-1 Figure 2-1 Simulations of stationary branching process that nevertheless test positive for *H*_poisson_ and $H_{\text{MR\_invalid}}$, respectively, owing to statistical fluctuations due to a relatively small sample size. Here we simulated a time series of length 13,000, similar to our longest datasets. Download Figure 2-1, TIF file.

We found a number of datasets that were positive for *H*_poisson_ or *H*_MR_invalid_. These tests were conducted to identify regimes of Poisson activity for which *r_k_* is expected to be a noisy fluctuation of ∼0, in which case the mean of *r_k_* would naturally not be significantly larger than zero, thereby being positive for the tests. In the datasets that tested positive for *H*_MR_invalid_ and *H*_poisson_; however, we observe the characteristic exponential decay in line with the prediction for a non-Poisson and noncritical branching process (0 < *m *<* *1), which effectively places these datasets outside of what the two tests are addressing. We therefore hypothesized that these datasets result from stationary branching processes, which are positive for either of the two tests because of fluctuations due to small sample size. This is supported by the observation that the estimated $\hat{m}$ for these datasets are consistent with the other accepted recordings. We then verified this by simulating subsampled stationary branching processes to the same time series length as our data and show that we could indeed observe these cases testing positive for either of the two tests (Extended Data Fig. 2-1). Hence, we chose to proceed with the datasets that either cleared all the stationarity tests or were positive for only *H*_MR_invalid_ or *H*_poisson_. We subsequently refer to these datasets as accepted datasets.

The accepted datasets were analyzed with the MR estimator, with the results also indicated in Figure 2. The dynamics of the habenula was treated as a branching process, such that the activity in the next timestep *A_t_*_+1_ is on average a linear function of that in the current timestep with the addition of some external drive with a mean rate *h*, as given in Equation 1. As the equation indicates, *m* encodes the amount of temporal correlation present in the system; the spatial correlation is implicit as this framework considers not individual units, but the total activity over the entire system. When measured in the timescale of neuron activity, *m *=* *0 denotes a state with no temporal correlation (i.e., asynchronous-irregular), while *m *=* *1 denotes a critical state where long-term temporal correlation is observed. When *m* is close to but not exactly 1 in the neuron activity timescale, the system is situated in a reverberating state that shares some of the benefits of the critical state without its drawbacks (Wilting and Priesemann, 2019b). As *m* is close to 1 in this regime, it is also characterized by long autocorrelation time.

**Figure 3.**
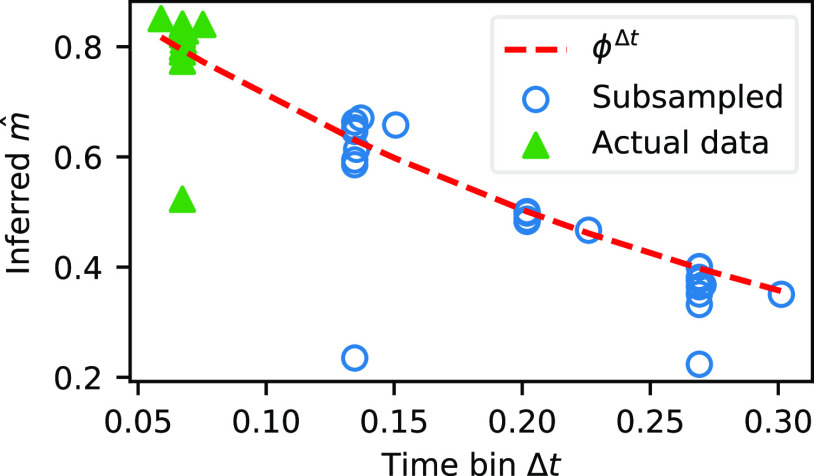
Inferred $\hat{m}$ values of the accepted datasets as well as temporally subsampled data. There is a systematic downward trend with increasing time bin Δ*t* size consistent with theoretical expectations. The theoretical fit (without the three outliers) is shown as a red dashed line. This trend is not by chance, as time-shuffled data indicate Poisson activity regardless of Δ*t* (Extended Data Fig. 3-1).

We emphasize that this reverberating state is assessed from *m* being close to 1 only when measured at the timescale of neural activity. When measured at slower timescales, the inferred value of *m* changes as it depends on the sampling rate. Specifically, data arising from stationary branching processes should display the branching activity in a systematic way across different timescales within the limits of its autocorrelation time. At different measurement time bins Δ*t*, the inferred branching parameter $\hat{m}$ at the time scale of Δ*t* should relate to the true branching parameter *m*_true_ at the actual timescale of the underlying process Δ*T* as follows:

(11) $$\hat{m}=m_{\text{true}}^{\Delta t/\Delta T}=\phi^{\Delta t},$$

where we have $\phi =m_{\text{true}}^{1/\Delta T}$ grouping the two unknown constants together as they cannot be estimated separately from data. We studied whether this relation holds for our datasets by temporally subsampling our recordings and rerunning the pipeline in each iteration (i.e., Δ*F*/*F* computation, MLspike inference, stationarity tests, and computation of $\hat{m}$). The results are given in Figure 3, where we show the inferred $\hat{m}$ values obtained from the accepted datasets alongside temporally subsampled data that pass the stationarity tests. Here we see a downward trend relative to increasing time bins that is generally consistent with Equation 11. This consistency supports the validity of the branching process and MR estimator in describing the dynamics in the habenula, as the change in $\hat{m}$ across different time bins is consistent with theoretical expectation. Excluding the three outliers, fitting Equation 11 to Figure 3 yields *ϕ* = 0.0326, which when converted to the timescale of Δ*T *=* *4 ms to reflect the true neural activity timescale (Wilting and Priesemann, 2019a) gives us $\hat{m}=0.986$, which is indeed in the reverberating regime. This is consistent with studies on mammalian cortex in the reverberating regime, which when measured at Δ*t *= 4 ms yielded $\hat{m}$ in the range of 0.963–0.998 with a median of 0.980 (Wilting and Priesemann, 2019a). On the other hand, Equation 11 also indicates what happens when the system is critical. As is to be expected, a critical system exhibits self-similarity across scales, and Equation 11 shows that when *m*_true_ = 1, $\hat{m}=1$ across different Δ*t*, which is not the case in the habenula. Lastly, we note that the systematic relationship shown in Figure 3 is not simply a product of chance, as the majority of the time-shuffled counterparts identically display Poisson activity with $\hat{m}=0$ (Extended Data Fig. 3-1), with the remaining few being positive for other nonstationarities.

10.1523/ENEURO.0287-21.2022.f3-1 Figure 3-1 A representative sample of the regression slopes *r_k_* at different time lags *k* for shuffled data. Each row is a specific dataset temporally subsampled to yield multiple effective sampling rates, which progressively becomes noisier as temporal subsampling reduces sample size. In each case, shuffling leads to a noisy horizontal line in the plot clustered at ∼0, indicating Poisson activity (*m *=* *0). Indeed, the majority test positive for *H*_poisson_, while the remaining few are positive for other nonstationarity tests. Download Figure 3-1, TIF file.

**Figure 4.**
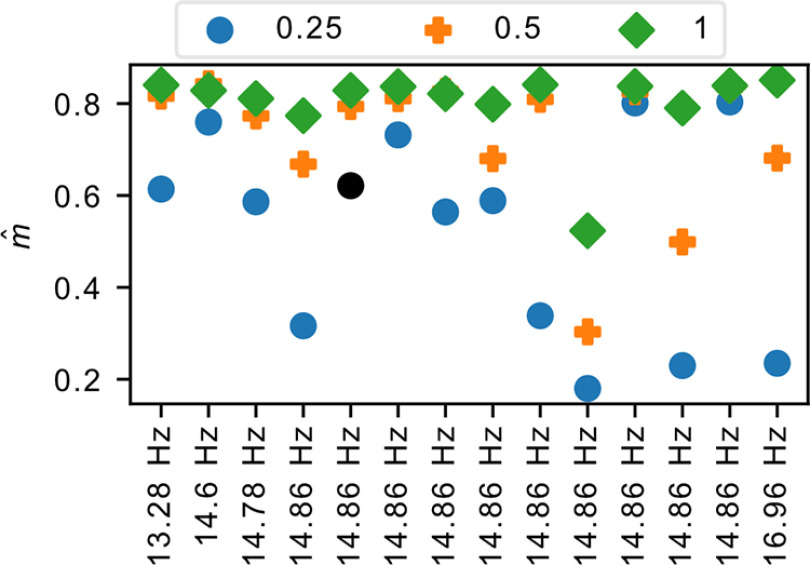
Effect of spatial subsampling on $\hat{m}$. The estimated branching parameter $\hat{m}$ for each dataset is labeled and ordered by its sampling rate at different subsample ratios. The one point in black failed a stationarity test. We see that, contrary to expectation, there appears to be systematic underestimation of $\hat{m}$ with spatial subsampling. We further investigate this in Extended Data Figure 4-1.

Since the estimator has been shown to be generally consistent over spatial subsampling in the cortex of mammals using electrical recordings (Wilting and Priesemann, 2018, 2019a), we tested whether this also holds here with calcium imaging data. Interestingly, we observe that to be not the case, as seen in Figure 4. To understand why, we simulated stationary branching processes at *m*_true_ = 0.985 and 0.9999, where the two cases reflect reverberating and critical dynamics, respectively. This is followed by temporal subsampling by a factor of 15, as neural activity timescale is approximately Δ*T *=* *4 ms (Wilting and Priesemann, 2019a); this temporal subsampling then brings the measurement timescale to be at Δ*t *=* *60 ms, which is close to that of our recordings (approximately Δ*t *=* *66–76 ms). In this coarser timescale, the effective values of *m* become *m *=* *0.7972 and 0.9985, respectively, as per Equation 11. We then performed spatial subsampling in two different ways. In the first case, we spatially subsample by binomial subsampling, where each spike has an equal probability of being “skipped” because of subsampling. We used this as a basis since MR estimator was proven to be consistent under this type of subsampling. In the second case, we follow actual experimental conditions where there is systematic subsampling of specific neurons. We do so by simulating a simple lattice network of neurons, each connected to four other neurons, and subsample by measuring only a random subset of the neurons. To mimic our experimental conditions, we first subsample the network to 250 neurons (approximately the number measured by our recordings) before investigating how further spatial subsampling affects the inferred $\hat{m}$ (similar to Fig. 4). We see in Extended Data Figure 4-1 that the MR estimator is consistent in all these cases, as expected.

10.1523/ENEURO.0287-21.2022.f4-1 Figure 4-1 Simulation results showing the impact of different types of spatial subsampling. In all cases, we simulated a system of 15,000 neurons before temporally subsampling by a factor of 15 and spatially subsampling to 250 neurons. Each subplot then shows the impact of further subsampling from this initial population of 250 neurons. ***A–H***, Left column (***A***, ***C***, ***E***, ***G***), Temporal subsampling by skipping bins; right column (***B***, ***D***, ***F***, ***H***), temporal summation by summing over skipped bins. ***A–D***, Spatial subsampling by binomial subsampling (i.e., skipping individual spikes). ***E–H***, Systematic subsampling by skipping neurons. ***A***, ***B***, ***E***, ***F***, *m*_true_ = 0.9999, *m *=* *0.9985; ***C***, ***D***, ***G***, ***H***, *m*_true_ = 0.985, *m *=* *0.7972. Download Figure 4-1, TIF file.

After verifying this, we reconsidered the process of temporal subsampling compared with our experimental conditions. Temporal subsampling is typically achieved by measuring a subset of the original data. When subsampling by a factor of *n*, for every *n* consecutive data points, we measure the *n*th element and discard the rest. The situation here, however, differs. Because of the slow decay time of gCaMP6s, MLspike would in principle be able to capture the spikes that occur in between time bins. In this case, then, for every *n* consecutive data points, we measure the sum over all *n* elements instead, resulting in a form of temporal summation. The right column of Extended Data Figure 4-1 shows the impact of this temporal summation. In most cases, we see that the MR estimator retains its consistency; however, we see in one case that it begins to systematically underestimate *m* with severe spatial subsampling. This is in fact the case that corresponds most with our setting: *m *=* *0.7972 (*m*_true_ = 0.985) with both temporal summation and systematic subsampling of neurons. The measured $\hat{m}$, therefore, serves as a lower bound to the true value, though we also note that between the subsampling factor of 0.5–1 (i.e., 125–250 neurons), which is approximately the range of neurons measured in our recordings), the measured $\hat{m}$ is still very close to the true value; severe underestimation only occurs when <100 neurons were measured. On the other hand, this underestimation also depends on the value of *m* in the measured timescale. In the case of *m *=* *0.9985 (*m*_true_ = 0.9999), *m* is close to 1, and we observe that the average effect of spatial subsampling is almost negligible, while the *m *=* *0.7972 (*m*_true_ = 0.985) case leads to a much more pronounced effect. This could be why this effect of spatial subsampling was not significantly observed in other studies where temporal summation may have been used, because in those cases data were collected using electrical recordings measured at a timescale of Δ*t *=* *4 ms, in which case the effective *m* is close enough to 1 that the effect of spatial subsampling remains small.

On the other hand, it was also noted that when very few neurons were measured, deviations could also come from heterogeneity within the network (Wilting and Priesemann, 2019a). Our simulations here consist of homogeneous neurons (since we used a lattice topology where every neuron is equivalent), indicating that systematic spatial subsampling can also have an effect on $\hat{m}$ estimation in homogeneous networks, provided temporal summation also occurred (which is quite commonly the case in neural data), although heterogeneity would naturally exacerbate the effect. Given the diversity of cell types in the habenula (Pandey et al., 2018), it is also likely that heterogeneity in the neuronal activity also contributed to the observed deviations on significant spatial subsampling.

These findings imply two things. First, our measured $\hat{m}$ is possibly a lower bound to the true value, although our simulations also indicated that at our measured population size, they should nevertheless remain close to the true value. Second, the true value is not at the critical point, as our simulations have shown that significant spatial subsampling should have only a negligible effect when *m* is critical, which is not observed in our data.

### Activity in the habenula has a high autocorrelation time

**Figure 5.**
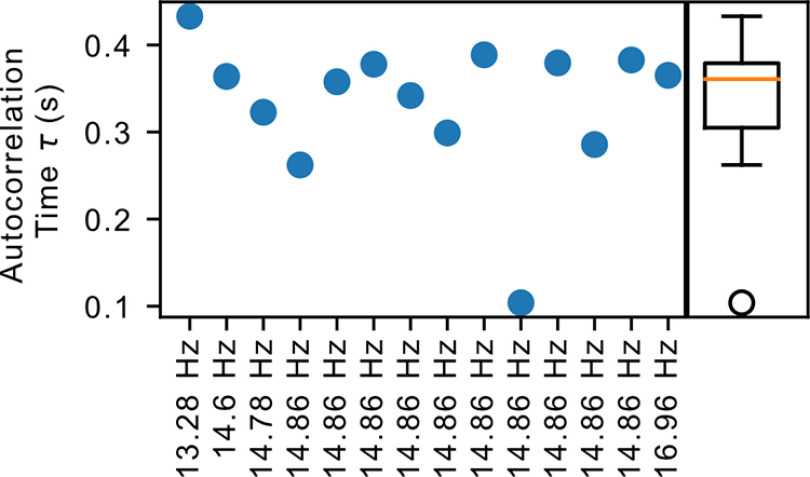
Autocorrelation time in the habenula. The autocorrelation time *τ* (in seconds) for each of the accepted datasets, labeled and sorted by the recording sampling rate. The box plot on the right shows the aggregated statistics of these values.

In the preceding section, we looked at how $\hat{m}$ changes with sampling rate and inferred that the system lies in a reverberating state by computing $\hat{m}$ in the neural activity timescale. Another way to assess the state of the system is to convert $\hat{m}$ into the autocorrelation time, which removes the dependence on sampling rate, thereby allowing a fair comparison between different measurement timescales while also providing a measure of persistence of information in the system. As the name suggests, the reverberating state displays long autocorrelation time where incoming information reverberates in the system before dissipating. This autocorrelation time *τ* is computed as *τ* = – Δ*t*/log *m* where Δ*t* is the measurement timescale (i.e., time bin; Wilting and Priesemann, 2018). Figure 5 shows the values of *τ* computed from the accepted datasets, which yielded consistent values ranging from 104 to 433 ms with a mean value of 333 ms (95% CI, 293, 374). The order of magnitude is consistent with *τ* measured from monkey prefrontal cortex and cat visual cortex, and is an order of magnitude smaller than the *τ* measured in rat hippocampus; the numerical results across these different systems were jointly reported to cover a range between 100 ms and 2 s with a median value at 247 ms (Wilting and Priesemann, 2019a).

### The habenula is reverberating and not critical

**Figure 6.**
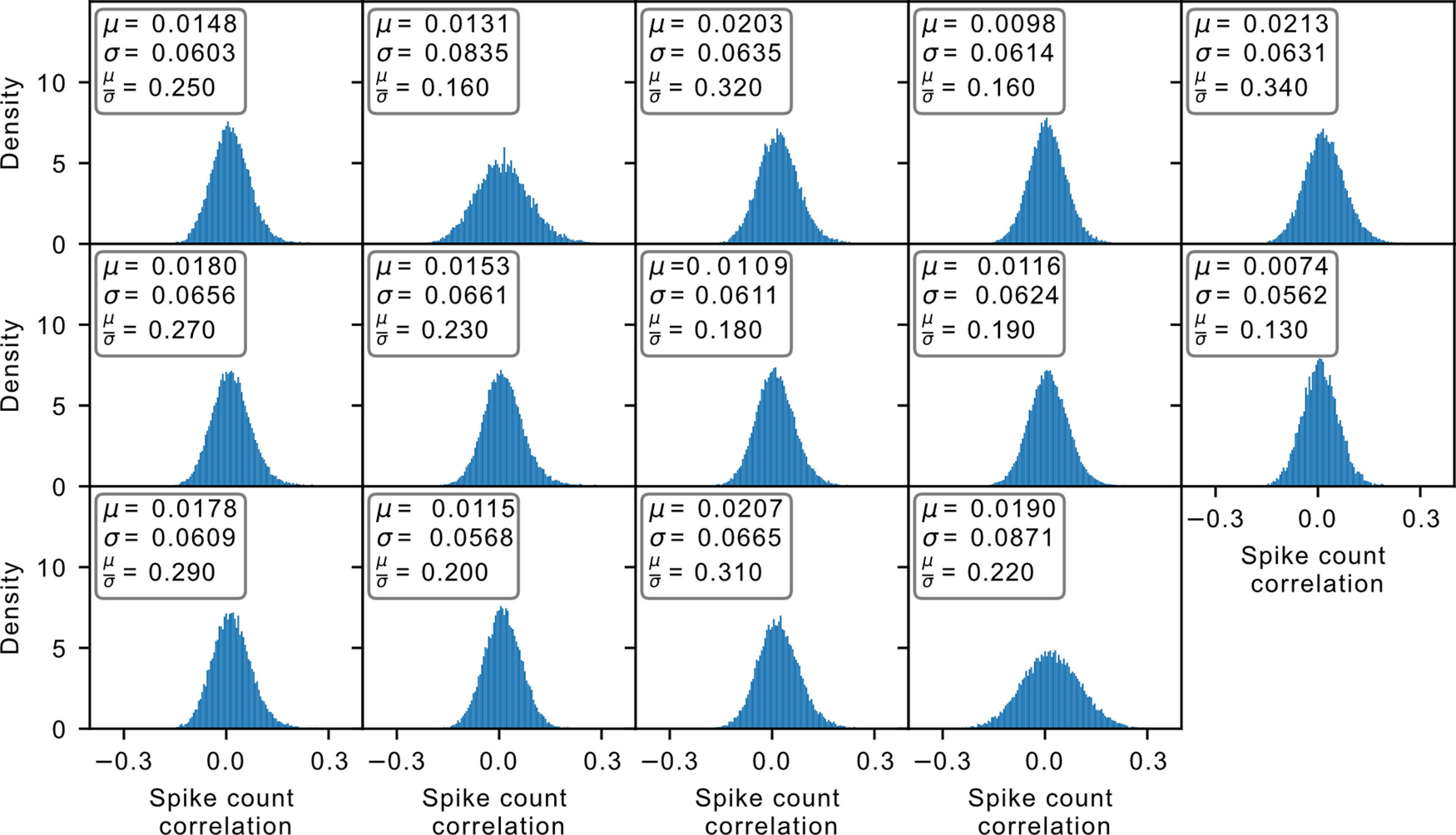
Spike count correlation distribution. Each panel indicates the distribution of a different dataset. For all the accepted datasets, the mean *μ* is significantly smaller than the width *σ* of the distribution. This finding is also consistent with further spatial subsampling of the system (Extended Data Fig. 6-1).

We have so far studied the system through the perspective of avalanche distributions and autocorrelation time in branching processes. We asked whether there is additional evidence for the habenula being in a reverberating rather than a critical state. It has been shown that for a state with critical avalanches (i.e., with branching parameter *m *=* *1), the spike count covariance between neurons in the system should be distributed with a mean value far from zero with a relatively small width (Dahmen et al., 2019). What we observe consistently in our system, however, are distributions that are largely symmetrical, ∼0 (Fig. 6, spike count correlation distribution, which normalized the support of the distribution to [–1, 1]). In our datasets, we found that the width is considerably larger than the mean. This finding remains consistent even with further spatial subsampling of the data (Extended Data Fig. 6-1). This is in fact more consistent with another dynamic regime known as the balanced state (Dahmen et al., 2019), which is characterized by an excess of inhibition.

10.1523/ENEURO.0287-21.2022.f6-1 Figure 6-1 Spike count covariance distribution of spatially subsampled data. Each row represents a specific dataset spatially subsampled by a factor given by the column. In all cases, we observe consistency in the relationship between the mean *μ* and the width *σ* of the distribution. Download Figure 6-1, TIF file.

**Figure 7.**
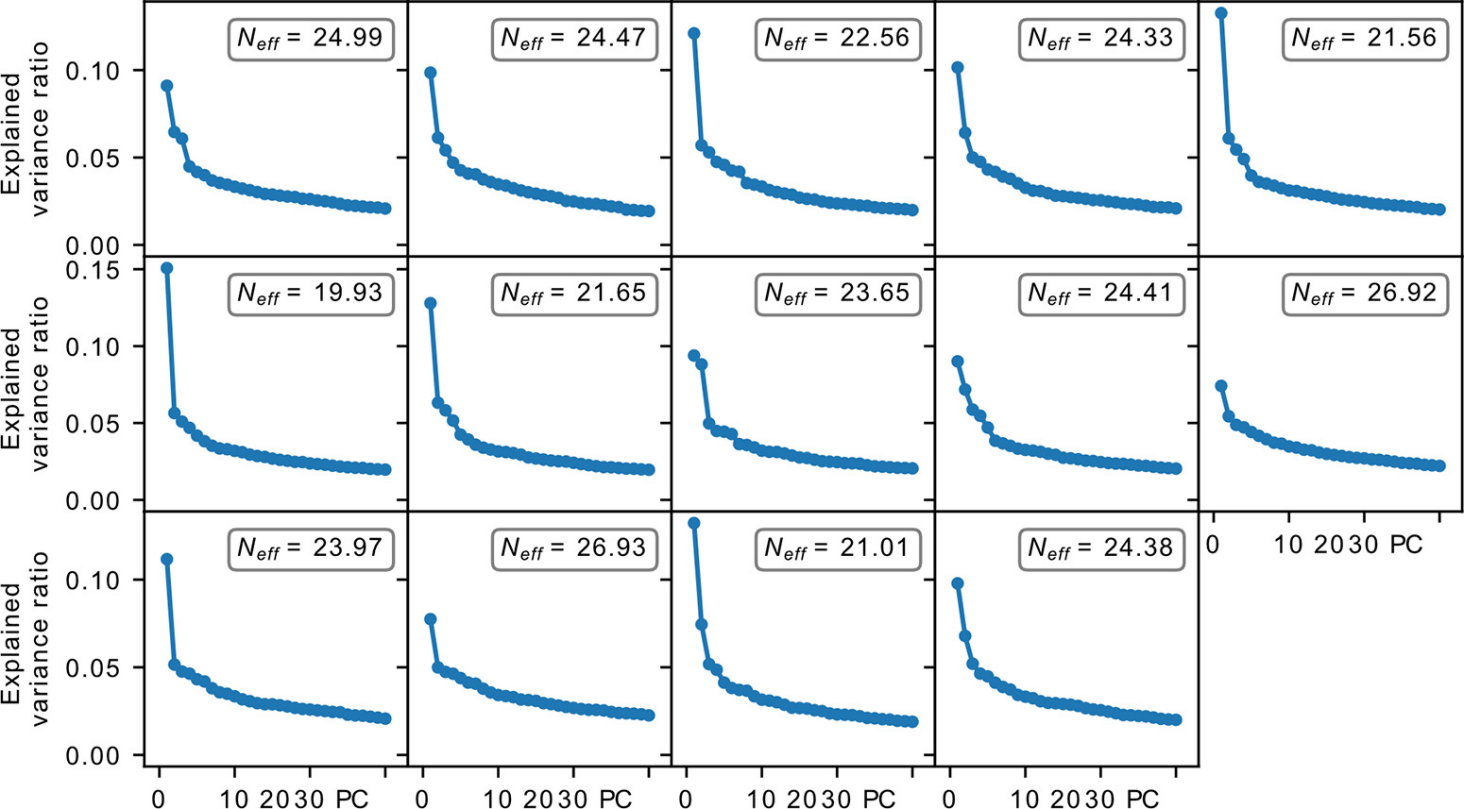
PCA explained variance ratio for the accepted datasets. All cases display a gradual decline instead of just a single dominant contribution, with the effective dimensions being significantly >1. In addition, activity along the different principal components have heterogeneous loadings (Extended Data Fig. 7-1). These deviate from what is expected from a neural system with critical avalanches.

**Figure 8.**
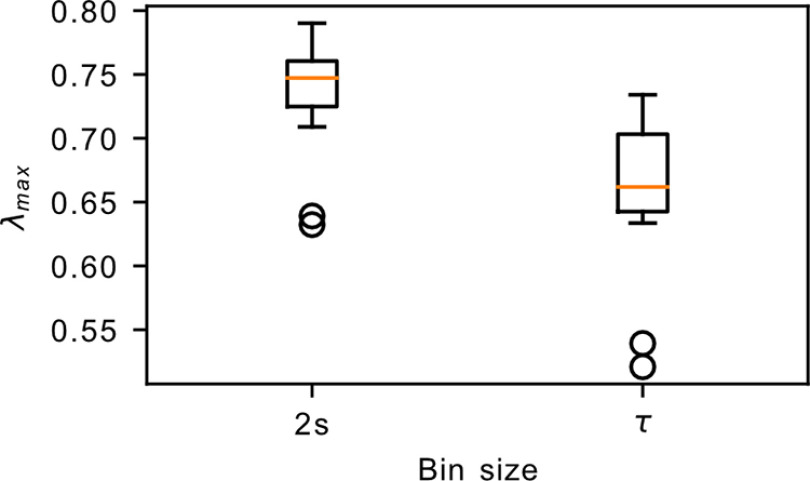
The largest eigenvalue *λ*_max_ of the connectivity matrix. The computation of *λ*_max_ requires the binning of the spikes into time bins. This plot shows the results obtained after binning the spikes into 2 s bins, as well as binning into different sizes for each dataset according to its own autocorrelation time *τ*. In both cases, the *λ*_max_ values lie well below the critical point at *λ*_max_ = 1.

We next looked into the dimensionality of the underlying dynamics. The dynamics of a critical avalanching system is characterized by a single dominant eigenvalue, leading to an effective dimensionality of ∼1, which can be probed by performing PCA on the spike count matrix of the system (Dahmen et al., 2019) with the effective dimension computed using the participation ratio (Abbott et al., 2011; Mazzucato et al., 2016). We observed that the habenula datasets display a more gradual decay in the explained variance ratio, which yields a larger effective dimension of ∼19.9–26.9 (Fig. 7). In addition, the activity along the different principal components are observed to have heterogeneous loadings (Extended Data Fig. 7-1), which contrasts the expected homogeneous loadings for a critical avalanching system (Dahmen et al., 2019). These observations, together with the preceding sections, strongly indicate that the habenula is not in a critical avalanching state. On the other hand, while the findings here may suggest that the habenula could reside in the dynamically balanced critical regime (the second type of criticality discussed in the study by Dahmen et al., 2019), our analysis revealed that the relevant measure *λ*_max_ lies significantly below the value that would be expected of such a regime (Fig. 8), indicating that the habenula is also not in the dynamically balanced critical state.

10.1523/ENEURO.0287-21.2022.f7-1 Figure 7-1 Loadings for the top three principal components. Each row represents a principal component, and each column is for a particular sample dataset, truncated to 50 neurons for clearer visualization. In all cases, the loadings are nonuniform with a mix of positive and negative contributions. Download Figure 7-1, TIF file.

## Discussion

The dynamics of a system influences how information is processed. Neural networks have in many instances been described as being either critical (Burns and Webb, 1976; Softky and Koch, 1993) or asynchronous (Van Vreeswijk and Sompolinsky, 1996; Brunel, 2000). The whole zebrafish brain has been suggested to operate at criticality, although regional differences were noted (Ponce-Alvarez et al., 2018). Here, we examined dynamics of the habenula *in vivo*, using calcium imaging at cellular resolution as a proxy for electrical activity. Through the analysis of avalanches, the autocorrelation time, and population statistics, we found no evidence of criticality, although the system is situated near criticality. And although it does display large fluctuation in population activity and long autocorrelation time, which are signatures of being near avalanche criticality, the habenula also bears features of asynchronous activity in the dynamically balanced state. On the one hand, the spike count covariance distribution of the asynchronous state has a small mean and a large width, in contrast to the large mean and small width of the avalanche critical state (Dahmen et al., 2019). Our analysis indicates that the habenula follows the former statistical behavior of asynchronous state. On the other hand, the asynchronous state is also characterized by weak fluctuations and short autocorrelation time on the population level, and this is where the habenula differs. Thus, our results suggest that the habenula is not exclusively critical or asynchronous.

Existing work on the asynchronous state revealed the existence of a second type of criticality representing the edge of chaos (Dahmen et al., 2019). Such a state is characterized, among others, by multidimensional activity that can be probed using PCA. Each mode of activity (projected to a particular principal component) is realized by a mixture of positive and negative neuron contributions of various magnitudes, which observe long autocorrelation times. In contrast, avalanche criticality has dimension typically ∼1, with a single dominant mode given by uniform contribution from all neurons. We observe signs of multidimensional dynamics in the habenula, although we have also shown that the habenula is not situated in this critical point of the second type. Together, our observations indicate that the habenula contains a mixture of characteristics from asynchronous and avalanche dynamics near their respective critical states.

There have been a number of attempts to reconcile the presence of both avalanche and asynchronous dynamics in a network. Simulations show that the incompatible asynchronous and avalanche dynamics can be maintained in the same neuronal network through a balance between excitatory (E) and inhibitory (I) signals at a specified average synaptic strength (Li and Shew, 2020). In other words, a mixture of avalanche and asynchronous neuronal dynamics can result depending on the relative strength of the excitatory and inhibitory synapses (i.e., the I/E weight ratio) and the average synaptic weight. At low I/E weight ratio and weak synaptic strength, the network is near the state of avalanche criticality; while at high I/E weight ratio and strong synaptic strength, it is at the heterogeneous asynchronous state (Ostojic, 2014) near the dynamically balanced critical regime (Dahmen et al., 2019). A system can exhibit characteristics of both avalanching and asynchronous dynamics at a crossover point with a proper balance of E and I (Li and Shew, 2020). This crossover point is not a critical point, which is consistent with what we observe in the habenula.

One interpretation of the dynamics observed is that the habenula is reverberating (Wilting and Priesemann, 2018), as indicated by the inferred *m* and autocorrelation time. This state is known to combine features from asynchronous and avalanche dynamics (Wilting and Priesemann, 2019a), which has also been observed here in the habenula. The reverberating state, which has been reported previously in mammalian cortical regions, balances the information processing benefits afforded by the critical state, such as sensitivity, but also reduces the drawbacks, such as lower specificity (Wilting and Priesemann, 2019b). A balance of the two may be more optimal for information processing (Wilting et al., 2018). In the reverberating state, small changes in parameter can lead to large changes in the computational properties of the network (Wilting et al., 2018), while the long autocorrelation time may serve as a form of working memory. This is important for adaptation; should there be a sudden change in the environment, the flexibility conferred by the reverberating state will enable optimal change in internal state and thus ensure survival.

## Synthesis

Reviewing Editor: Niraj Desai, National Institute of Neurological Disorders and Stroke Decisions are customarily a result of the Reviewing Editor and the peer reviewers coming together and discussing their recommendations until a consensus is reached. When revisions are invited, a fact-based synthesis statement explaining their decision and outlining what is needed to prepare a revision will be listed below. The following reviewer(s) agreed to reveal their identity: Emre Yaksi.

Thank you for revising your manuscript in light of the first round of reviews. Your revised manuscript has now been read by two expert reviewers, whose comments are below. Both believe - as do I - that the manuscript has considerable merit. However, we all also believe that several points must be revisited and clarified before the manuscript can be accepted for publication. These points are described in detail in the two reviews appended below to this message. Please respond to them point by point.

REVIEW #1

General

This study aims to determine the underlying network dynamics of the habenula, an evolutionarily conserved structure, by analyzing population level recordings at single cell resolution via 2-photon imaging in resting zebrafish larvae. The authors utilize a simple measure m, the branching ratio, which characterizes the evolution of the network activity along the spectrum of exponential decay to exponential growth. They acknowledge the limitations of their methods (spatial subsampling) and in turn leverage a previously published multistep regression estimator (Wilting and Priesemann, 2018) to more accurately estimate the branching ratio. They determine, through these estimated values of m (and several other measures), that the habenula is in a reverberating state which shares benefits of the critical state while being free of some drawbacks associated with it. By demonstrating this in vivo, these findings would give weight to previous work suggesting the brain might naturally be in this reverberating state.

Specific

I appreciate that the authors carefully test each dataset for stationarity and the multiple measures used to test the dynamical state are stated clearly and cover a wide range of analyses. However, I cannot recommend this manuscript for publication in its current state.

Major comments:

• Fundamental methods and results statistics are missing. For instance: How many planes on average were recorded in each volume stack? Were all movie pre-processing steps carried out using suite2p? Were ROIs manually curated? How many ROIs/recording? What sort of spike rate distributions and network correlations were observed? Are these in agreement with previously published results?

• What is the reasoning/citation behind neurons with fluorescence z-score>2 to be considered active? I’m concerned that this definition combined with the long tail of the indicator leads to a large number of spuriously active bins in your raster (Fig. 1D) and possible errors in any downstream analysis.

• From what I understand, power law statistics were computed from one 45-second recording for each (N=2) larvae. This doesn’t seem enough to collect proper statistics to conduct avalanche analysis. How many avalanches were obtained from each animal after processing via the powerlaw package? Was any sort of avalanche shape analysis conducted?

Minor comments:

• 132-133: “time-step between images in the same plane was 0.18 sec, although there were delays of 1.74 sec every 3 stacks” - These numbers are slightly confusing. Is the imaging rate ∼5.5 Hz or ∼1.7 Hz and how do either of these line up with your reported imaging rates of 13-17 Hz? This part of your methods needs clarification.

• Data is analyzed via z-scored fluorescence, non-negative deconvolution (suite2p), and spike deconvolution (MLspike). Is there a reason why discretized spikes (MLspike) could not be used for all analyses?

• Adjust y-axis of Fig. 3 (0.95<y<1.01) to minimize white space and show any underlying trends in data.

• Fig. 6 x-labels seem to be misplaced on some sub-figures.

REVIEW #2

In this short manuscript the authors investigated the features of spontaneous activity in zebrafish habenula, and argue that habenula is neither fully asynchronous nor perfectly critical, but is reverberating. Later authors discussed how this reverberating state is important for enabling high flexibility y of habenular activity/tuning, thus providing a potential mechanism for the optimal predictions in a changing environment.

In general, I find this manuscript very interesting and exciting, yet somewhat rather preliminary and not well presented. Most of the presented analysis are not well justified or explained in the results section. Moreover, I also find that in several places the authors over interpreted some of their results. A major example of such over interpretation is the concept of asynchronous activity, as argued by the authors. I find it rather difficult to interpret mean and variance results, and come up with the argument of asynchronous activity. If the authors wish to make this a stronger claim, I think they need to better evaluate the synchrony/asynchrony in their system. Similarly, the arguments about criticality and avalanches are rather tangential and based on very few measures, which are also not very well justified throughout the text.

Overall, I find this study very interesting, but in its current form It appears to be rather speculative and many claims are not well justified, by backing up with extensive analysis (criticality/synchrony/reverberations) or data (e.g. predictions, plasticity etc). I will be excited to review a revised version of this paper, and I really hope that the authors will do a better job in presenting their results (especially results section will need better presentation and justification of the selected analysis and their interpretations) and supporting their arguments with additional and well justified analysis.

## Author Response

We thank the reviewers for their helpful comments, which have allowed us to improve this work further. Before we address the comments point-by-point, we would like to highlight the significant changes made in the manuscript, as well as the underlying reasons.

In Fig. 1, we previously used a 1.44 Hz volumetric recording to show that the population activity in the habenula does not have silent time bins. We noted that the microscope used had some systematic delay when volumetric imaging was performed. This could not be eliminated by upgrading the hardware or software. In addition, our previous way of inferring activity for this recording was somewhat arbitrary, taking zscore >= 2 as a measure of activity. We emphasize that this thresholding had been carried out only for the volumetric recording in the previous Fig 1; all subsequent sections and analyses do NOT include this step. This was done previously because MLspike, our primary approach for inferring discrete spikes, does not work reliably for data with very slow sampling rate. In this revised version, we sidestep all the problems and potential confusion by replacing this with our other datasets at ∼15 Hz (16 recordings), which allows us to directly probe for silent time bins through the spikes inferred by MLspike. We found all 16 recordings did not display any silent time bins. While these ∼15 Hz recordings are single plane, the lack of silent time bins implies an absence of silent time bins over the entire habenula. The conclusion is therefore unchanged.

The analyses in Fig. 2 onwards were previously carried out using deconvolved calcium traces computed using suite2p. This was done because the earlier iteration of this work included slow volumetric recordings (1-2 Hz) that could not be analysed reliably by MLspike owing to the slow sampling rate. However, since we have now excluded the slow recordings due to the delays, we can now perform the complete analyses solely using MLspike. This is a better measure of activity as it is in terms of discrete spikes, while calcium deconvolution returns a non-absolute measure of activity. In addition, using discrete spikes leads to much better correspondence with results in the literature, which were computed using discrete spikes as a basis (though in their case, spikes were directly measured using electrical equipment).

REVIEW #1

General

This study aims to determine the underlying network dynamics of the habenula, an evolutionarily conserved structure, by analyzing population level recordings at single cell resolution via 2-photon imaging in resting zebrafish larvae. The authors utilize a simple measure m, the branching ratio, which characterizes the evolution of the network activity along the spectrum of exponential decay to exponential growth. They acknowledge the limitations of their methods (spatial subsampling) and in turn leverage a previously published multistep regression estimator (Wilting and Priesemann, 2018) to more accurately estimate the branching ratio. They determine, through these estimated values of m (and several other measures), that the habenula is in a reverberating state which shares benefits of the critical state while being free of some drawbacks associated with it. By demonstrating this in vivo, these findings would give weight to previous work suggesting the brain might naturally be in this reverberating state.

Specific

I appreciate that the authors carefully test each dataset for stationarity and the multiple measures used to test the dynamical state are stated clearly and cover a wide range of analyses. However, I cannot recommend this manuscript for publication in its current state.

Major comments:

• Fundamental methods and results statistics are missing. For instance: How many planes on average were recorded in each volume stack? Were all movie pre-processing steps carried out using suite2p? Were ROIs manually curated? How many ROIs/recording? What sort of spike rate distributions and network correlations were observed? Are these in agreement with previously published results?

As we have now removed the volumetric recording from this work, the remaining recordings are all single plane. The pre-processing steps for the recordings were indeed all carried out using suite2p, and subsequently the ROIs identified by suite2p were manually curated to exclude neurons outside the region of the habenula. Over the different recordings we have 152-252 ROIs with the mean at 205.6. This information is provided in the revised Methods section.

We averaged the spiking activity of each neuron to compute the spike rate distribution for each recording (Fig. 1-1) and in each case they generally range between 0 to 5 Hz. Fig. 1C shows that the mean and SD of the distributions are quite similar across recordings, respectively averaging across recordings at 2.91 Hz and 0.94 Hz. The spike rate distributions agree with previously published results (https://www.sciencedirect.com/science/article/pii/S0896627317300351).

We are able to detect clusters of activity, similar to what was previously described (e.g. Jetti, S. K., Vendrell-Llopis, N. & Yaksi, E. Current biology 24, 434-439 (2014)), as shown in the examples below:

However, these clusters are only prominent when we used wide sliding windows in our computation of the fluorescence baseline, suggesting that a significant portion of the clusters may not actually co-occur in time, but are instead in close temporal proximity. In any case, we do not segregate our data into clusters in this work for two main reasons:

Our aim is to analyse the population activity of the habenula as a whole. Clustering the neurons beforehand, then, would not address this aim. The existence of different clusters also do not pose any issue in the population analysis, as the emergent population dynamics may also be in part due to the interaction between the different clusters.

As our analyses are restricted to single plane recordings (with around 150-250 neurons), each cluster consists of a small number of neurons. Applying statistical analyses to these small clusters may lead to difficulties in interpreting the results with confidence. In our opinion, these studies would be better left for when fast volumetric recordings are available.

• What is the reasoning/citation behind neurons with fluorescence z-score>2 to be considered active? I’m concerned that this definition combined with the long tail of the indicator leads to a large number of spuriously active bins in your raster (Fig. 1D) and possible errors in any downstream analysis.

In the previous manuscript, this thresholding procedure was only done as a rough measure of activity for the volumetric recording in Fig 1 alone. All subsequent analyses did not include this step. We have now excluded the slow recording, which allows us to remove this thresholding step entirely as we could use MLspike.

• From what I understand, power law statistics were computed from one 45-second recording for each (N=2) larvae. This doesn’t seem enough to collect proper statistics to conduct avalanche analysis. How many avalanches were obtained from each animal after processing via the powerlaw package? Was any sort of avalanche shape analysis conducted?

We have now used recordings that are 10 minutes long. These respectively yielded 360 and 207 avalanches. We have now included further avalanche analysis to support our claim of non-criticality. Specifically, we looked at both shape collapse (Fig 1-2), and the scaling relation between the shape collapse parameter and the exponent for the mean avalanche size given duration function (equation (8)). Experimental studies have shown agreement with theory that neural avalanches are parabolic in shape, but this was not observed here. In addition, equation (8) was not satisfied (and was in fact off by a large margin), again supporting the claim that the habenula is not critical.

Minor comments:

• 132-133: “time-step between images in the same plane was 0.18 sec, although there were delays of 1.74 sec every 3 stacks” - These numbers are slightly confusing. Is the imaging rate ∼5.5 Hz or ∼1.7 Hz and how do either of these line up with your reported imaging rates of 13-17 Hz? This part of your methods needs clarification.

This delay only exists for the volumetric recordings (1-2 Hz). It is absent in single-plane recordings. Since we have removed the use of volumetric recording in Fig 1, this part has now been removed.

• Data is analyzed via z-scored fluorescence, non-negative deconvolution (suite2p), and spike deconvolution (MLspike). Is there a reason why discretized spikes (MLspike) could not be used for all analyses?

We previously used both methods because we included analysis of slow volumetric recordings, which MLspike could not be used for. We agree that in the current state of the work MLspike would be more appropriate, and have now changed all the analyses to use MLspike results instead. MLspike is preferable as it is a direct measure of spiking activity while deconvolution is both a non-absolute (due to an unknown scaling factor) and indirect (as it estimates calcium influx (Stringer & Pachitariu, 2019)) measure of activity. Also, this allows us to directly compare with results in literature on cortical systems which use measured spikes as a basis. We see in the manuscript that there is now close correspondence.

Reference:

Stringer, C., & Pachitariu, M. (2019). Computational processing of neural recordings from calcium imaging data. Current opinion in neurobiology, 55, 22-31.

• Adjust y-axis of Fig. 3 (0.95<y<1.01) to="” minimize="” white="” space="” and="” show="” any="” underlying="” trends="” in="” data.<br="">The current results with MLspike no longer has this extra white space (now in Fig 4).

• Fig. 6 x-labels seem to be misplaced on some sub-figures.

This has been fixed.

REVIEW #2

In this short manuscript the authors investigated the features of spontaneous activity in zebrafish habenula, and argue that habenula is neither fully asynchronous nor perfectly critical, but is reverberating. Later authors discussed how this reverberating state is important for enabling high flexibility y of habenular activity/tuning, thus providing a potential mechanism for the optimal predictions in a changing environment.

In general, I find this manuscript very interesting and exciting, yet somewhat rather preliminary and not well presented. Most of the presented analysis are not well justified or explained in the results section. Moreover, I also find that in several places the authors over interpreted some of their results. A major example of such over interpretation is the concept of asynchronous activity, as argued by the authors. I find it rather difficult to interpret mean and variance results, and come up with the argument of asynchronous activity. If the authors wish to make this a stronger claim, I think they need to better evaluate the synchrony/asynchrony in their system.

We agree that the spike count covariance distributions could be difficult to interpret as it is scale dependent. Previously we reported spike count covariance distributions as these quantities are required to compute λ_max in Fig. 8, which is to determine whether or not the system is in the dynamically balanced critical state (another type of critical state different from avalanche criticality). While this calculation remains unchanged, we now plot Fig 6 using spike count correlation instead, which normalizes the range to within [-1,1] to aid interpretation. We now see that normalized in this way, the spike count correlation is centered very near zero with its width larger than the mean.

We highlight that while this is one of the characteristics of the balanced state with asynchronous activity (Dahmen et al. 2019), we are not suggesting that the habenula exists in that state. Indeed, in the perspective of the MR estimator, the asynchronous state has m=0, which is not what we have observed in the habenula. Instead, what we are saying is that the habenula appears to exhibit some properties of this dynamically balanced, asynchronous state (such as the spike count correlation distribution and the dimension of the activity). In the discussion section, we consider previous work that show the possibility of neural systems concurrently exhibiting properties of these different dynamical states.

In relation to our central claim, what these findings do suggest is that the habenula is not in the critical avalanching state, as the work by Dahmen et al. has shown that the spike count covariance (and therefore correlation) should be centered far from zero with its width smaller than the mean if the system is in the critical avalanching state.

Similarly, the arguments about criticality and avalanches are rather tangential and based on very few measures, which are also not very well justified throughout the text.

We added more analyses in the revised manuscript. Overall, our central proposal is that the habenula is not in the critical avalanching state, but instead in the reverberating state. The different sections in our work seeks to find signs of avalanche criticality in different ways, and in each case we do not see any strong positive sign of such a state. We summarize our findings in relation to this below.

Avalanche analysis

The critical avalanching state is naturally best analyzed through avalanches. We begin by showing that 10+ Hz recordings do not yield avalanches due to the absence of silent time bins, therefore we worked with 100+ Hz data for this analysis. We see the absence of power law distributions in the avalanche size and duration. Avalanche shape collapse serves as an additional test, where in our case we do observe collapse but of a different shape (both theory and experiment on critical neural systems show parabolic shapes,but we observe flat horizontal lines). We probed further using scaling relation (8), which is also not satisfied. There is at least one more commonly used scaling relation to test for criticality, but it could not be done here as it requires the critical exponents from the power law distributions in avalanche size and duration. We highlight that claiming criticality requires agreement across many tests, among which power law distributions in avalanche size and duration normally serve as a starting point. We see in our system that these distributions do not fit a power law, and together with further analyses of avalanche shape collapse and scaling relation (8), we find no strong support for criticality.

MR estimation

We recognized that the non-critical avalanche results could be due to spatial subsampling. We then turned towards an estimator known to work consistently under spatial subsampling (specifically, binomial subsampling). While we do subsequently see that the MR estimator underestimates the true value under systematic neural subsampling (Fig. 4 and 4-1), we nevertheless showed that a difference in this underestimation exists between a critical and reverberating system, and our results on the habenula corresponds to the latter. This again supports the claim that the habenula is not critical, and through the MR estimator, we show that it is in fact near, though not at criticality, in a regime known as the reverberating regime. A comparison with recent studies on mammalian cortical systems show agreement.

Spike count covariance

We lastly added further supporting evidence from other approaches, which is through spike count covariance/correlation as well as the dimension of the population activity. The work of Dahmen et al. (2019) show that a critical avalanching system has spike count covariance with mean far from zero and width smaller than the mean with the effective dimension of the activity being very near 1. Our computations on the habenula, on the other hand, deviate from the above expectation for a critical avalanching system.

Since this analysis by Dahmen et al. was designed to infer what they call criticality of the second kind (i.e. the dynamically balanced critical state, which is distinct from the more commonly studied critical avalanching state), we extended our analysis to cover that possibility as well. A defining measure for this state, is λ_max, which is 1 when the system is in this dynamically balanced critical state. For the habenula we observe this quantity to be far from 1, indicating that it is also not in this critical state.

Overall, through these three different approaches (avalanche analysis, MR estimator, spike count covariance), we do not observe any significant support for criticality, and therefore conclude that the habenula is not in this state. Our explorations, however, show that the habenula does exist in a meaningful dynamical state, which in this case is the reverberating regime. This does have some benefits of the critical state but without some of its drawbacks.

Overall, I find this study very interesting, but in its current form It appears to be rather speculative and many claims are not well justified, by backing up with extensive analysis (criticality/synchrony/reverberations) or data (e.g. predictions, plasticity etc). I will be excited to review a revised version of this paper, and I really hope that the authors will do a better job in presenting their results (especially results section will need better presentation and justification of the selected analysis and their interpretations) and supporting their arguments with additional and well justified analysis.

The main conclusion is that habenula spontaneous activity has the dynamics of a reverberating system. This is supported by the analysis described above. The framework of predictive coding is not central to this paper and has been removed. The notion that the habenula has a role in behavioural flexibility is based on published data (see citations in the second paragraph of the Introduction). The aim of this paper is to examine the dynamics of the habenula, as this can provide insight into how this flexibility is achieved.
