# Supplementary figures and images for: Dynamics and Potential Significance of Spontaneous Activity in the Habenula
Source: eNeuro. 2022 Sep 1;9(5):ENEURO.0287-21.2022. doi: 10.1523/ENEURO.0287-21.2022 (PMC9450562; doi:10.1523/ENEURO.0287-21.2022)

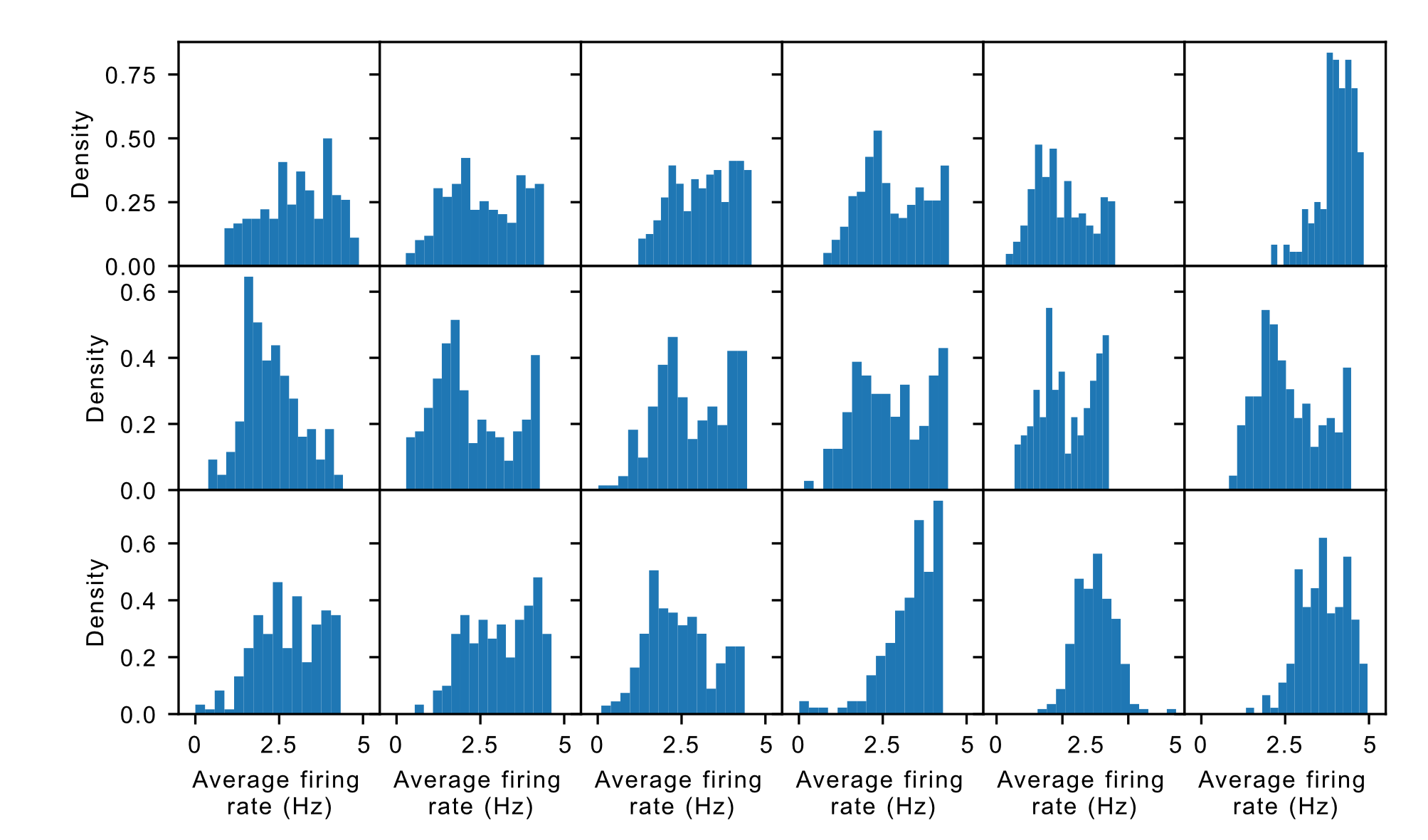

Supplement: Figure 1-1 — Average firing rate distributions for each recording computed from the discrete spikes inferred using MLspike. Download Figure 1-1, TIF file. [file enu-eN-NWR-0287-21-s02.tif]

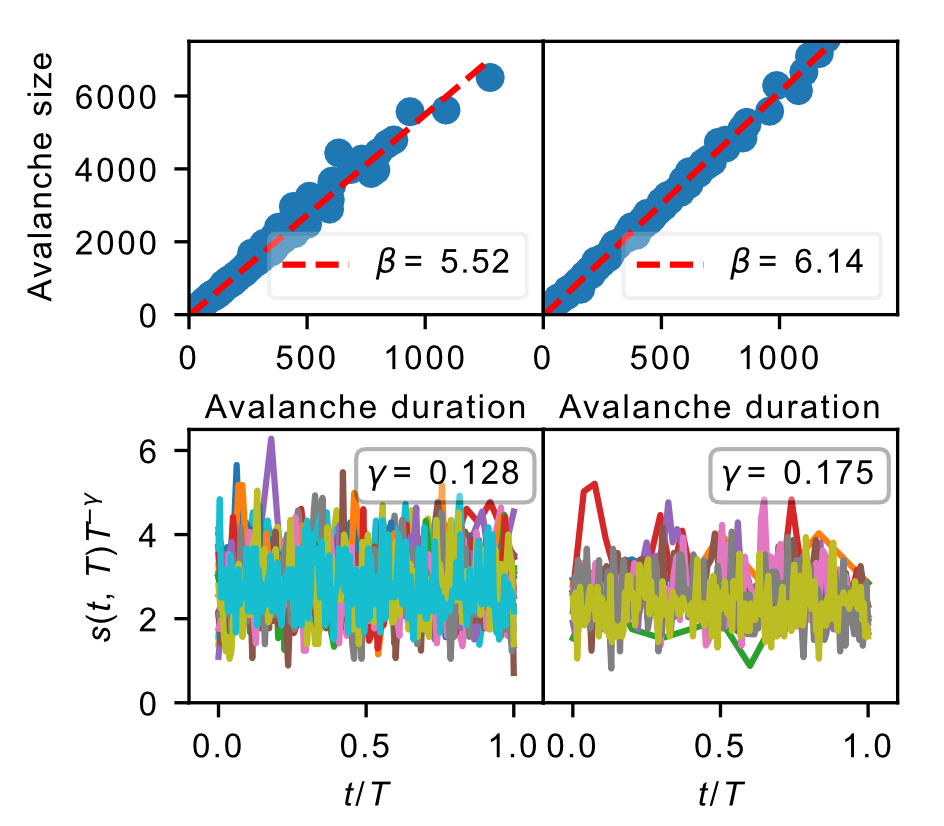

Supplement: Figure 1-2 — Further avalanche analysis. Top row, Mean avalanche size given duration. Bottom row, Avalanche shape collapse. We see that β and γ are far from satisfying Equation 10, further indicating that the habenula is not critical. Download Figure 1-2, file. [file enu-eN-NWR-0287-21-s03.tif]

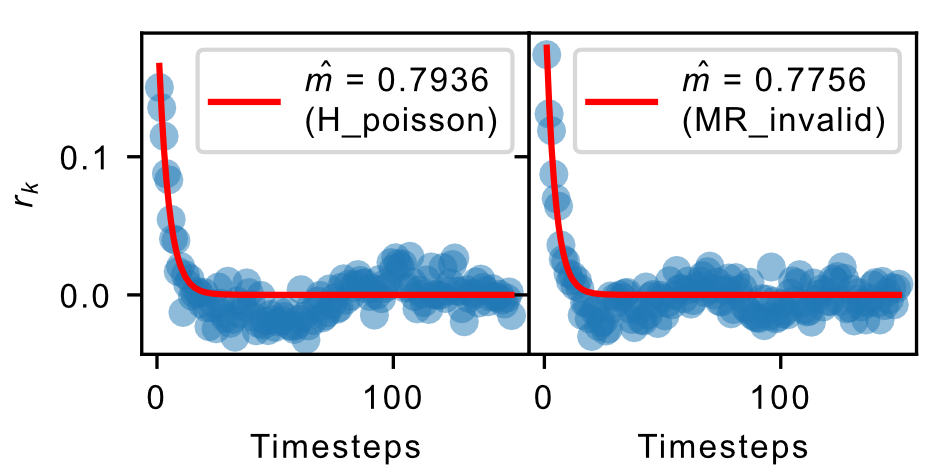

Supplement: Figure 2-1 — Simulations of stationary branching process that nevertheless test positive for Hpoisson and HMR_invalid, respectively, owing to statistical fluctuations due to a relatively small sample size. Here we simulated a time series of length 13,000, similar to our longest datasets. Download Figure 2-1, TIF file. [file enu-eN-NWR-0287-21-s04.tif]

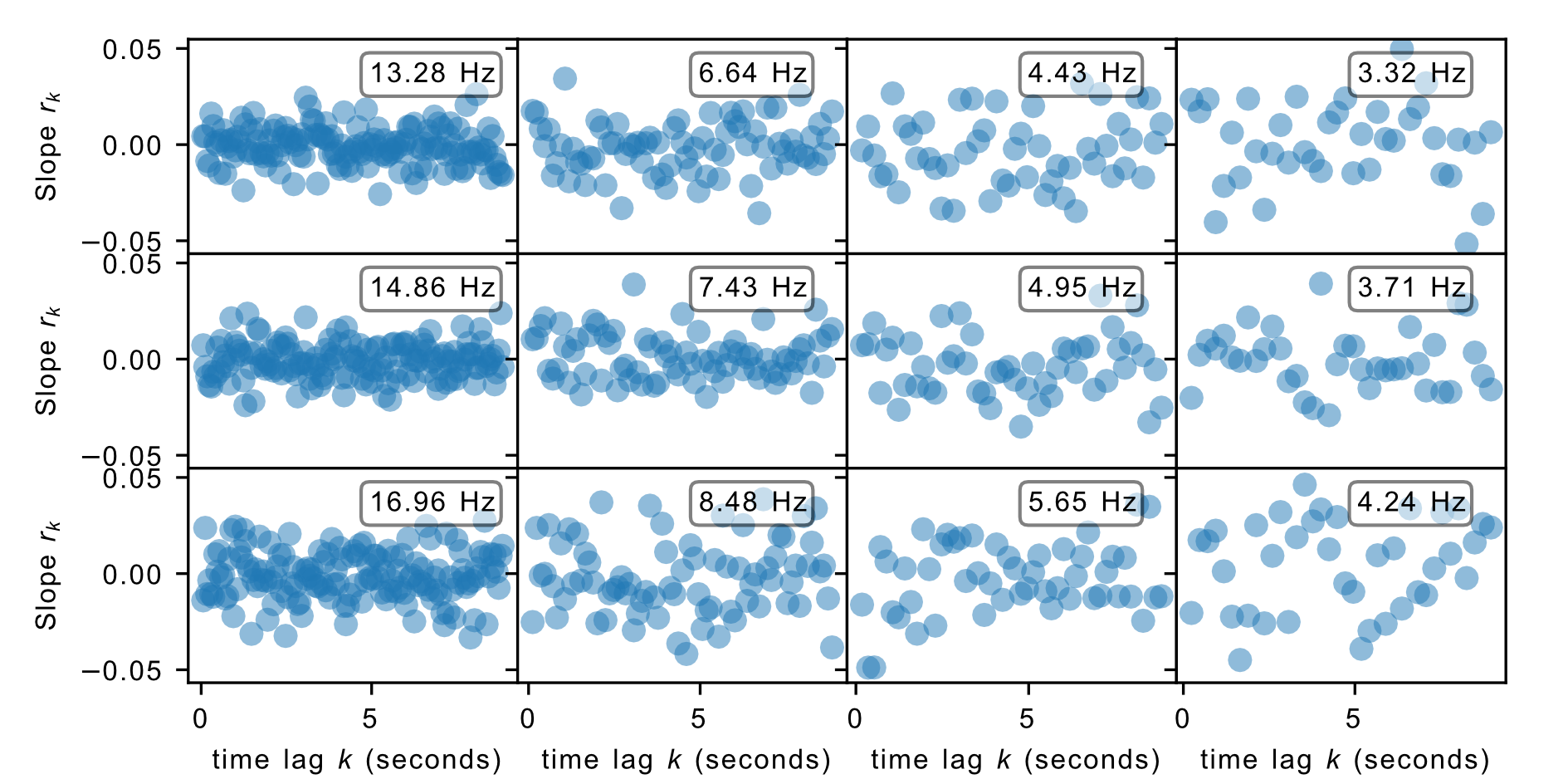

Supplement: Figure 3-1 — A representative sample of the regression slopes rk at different time lags k for shuffled data. Each row is a specific dataset temporally subsampled to yield multiple effective sampling rates, which progressively becomes noisier as temporal subsampling reduces sample size. In each case, shuffling leads to a noisy horizontal line in the plot clustered at ∼0, indicating Poisson activity (m = 0). Indeed, the majority test positive for Hpoisson, while the remaining few are positive for other nonstationarity tests. Download Figure 3-1, TIF file. [file enu-eN-NWR-0287-21-s05.tif]

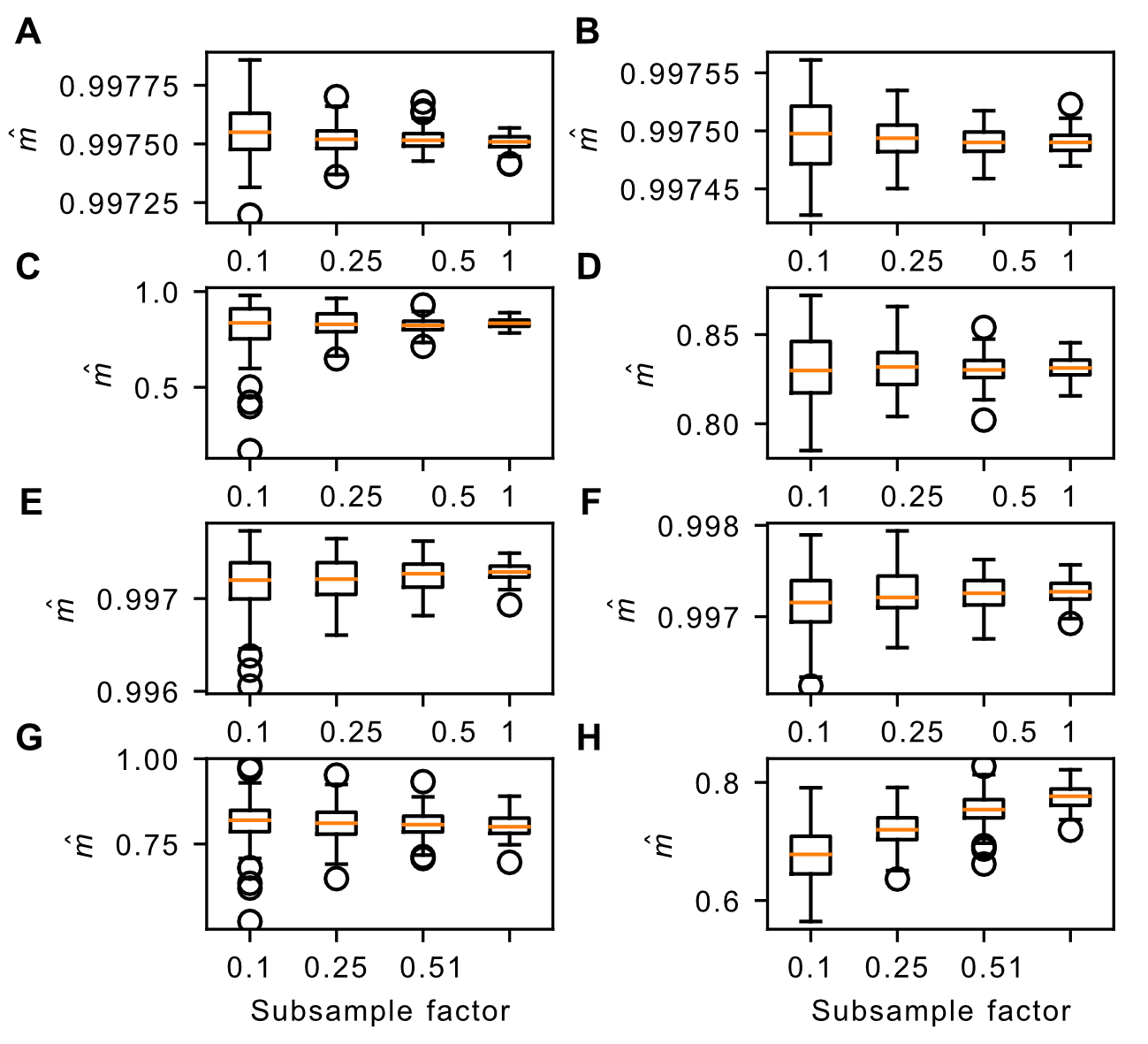

Supplement: Figure 4-1 — Simulation results showing the impact of different types of spatial subsampling. In all cases, we simulated a system of 15,000 neurons before temporally subsampling by a factor of 15 and spatially subsampling to 250 neurons. Each subplot then shows the impact of further subsampling from this initial population of 250 neurons. A–H, Left column (A, C, E, G), Temporal subsampling by skipping bins; right column (B, D, F, H), temporal summation by summing over skipped bins. A–D, Spatial subsampling by binomial subsampling (i.e., skipping individual spikes). E–H, Systematic subsampling by skipping neurons. A, B, E, F, mtrue = 0.9999, m = 0.9985; C, D, G, H, mtrue = 0.985, m = 0.7972. Download Figure 4-1, TIF file. [file enu-eN-NWR-0287-21-s06.tif]

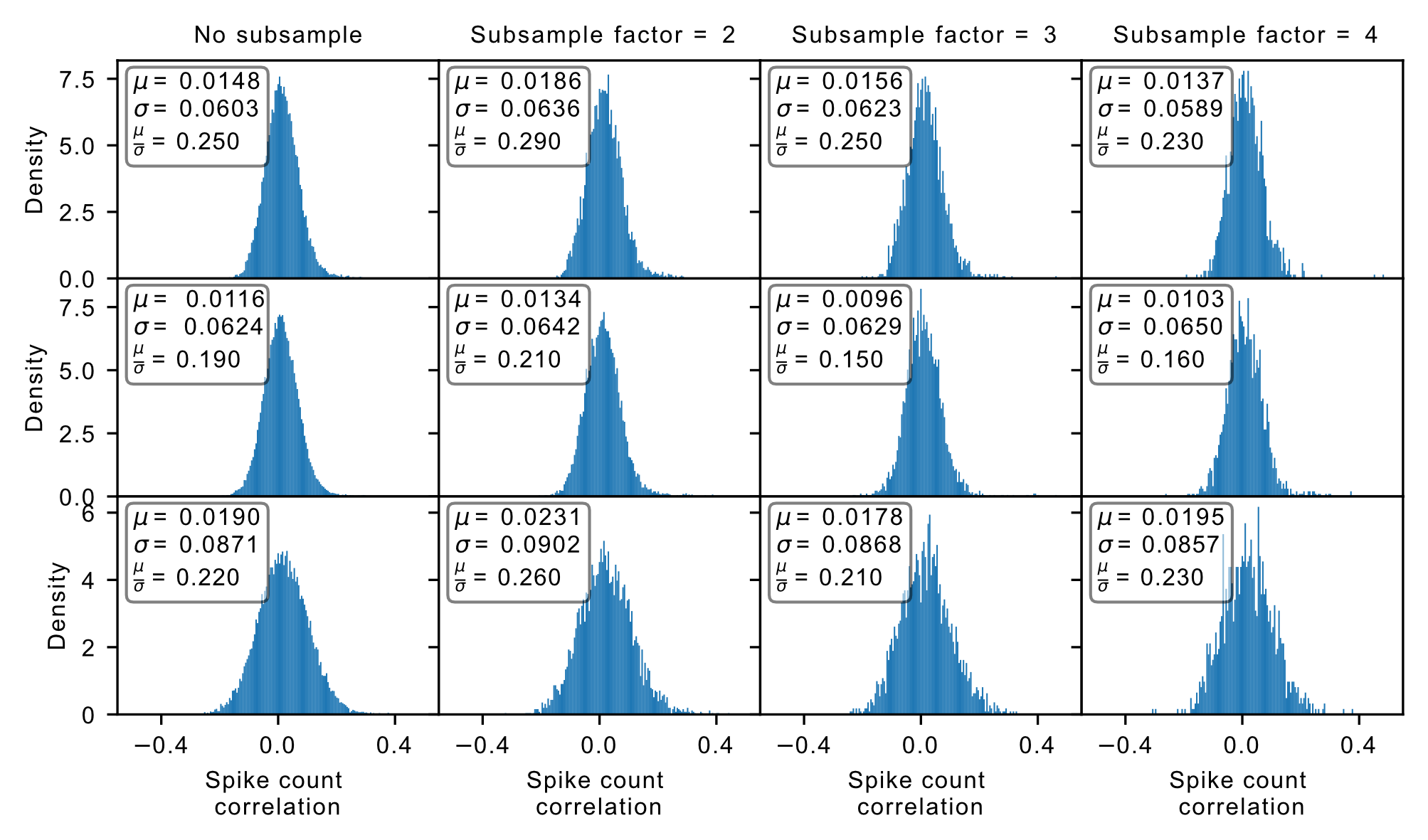

Supplement: Figure 6-1 — Spike count covariance distribution of spatially subsampled data. Each row represents a specific dataset spatially subsampled by a factor given by the column. In all cases, we observe consistency in the relationship between the mean μ and the width σ of the distribution. Download Figure 6-1, TIF file. [file enu-eN-NWR-0287-21-s07.tif]

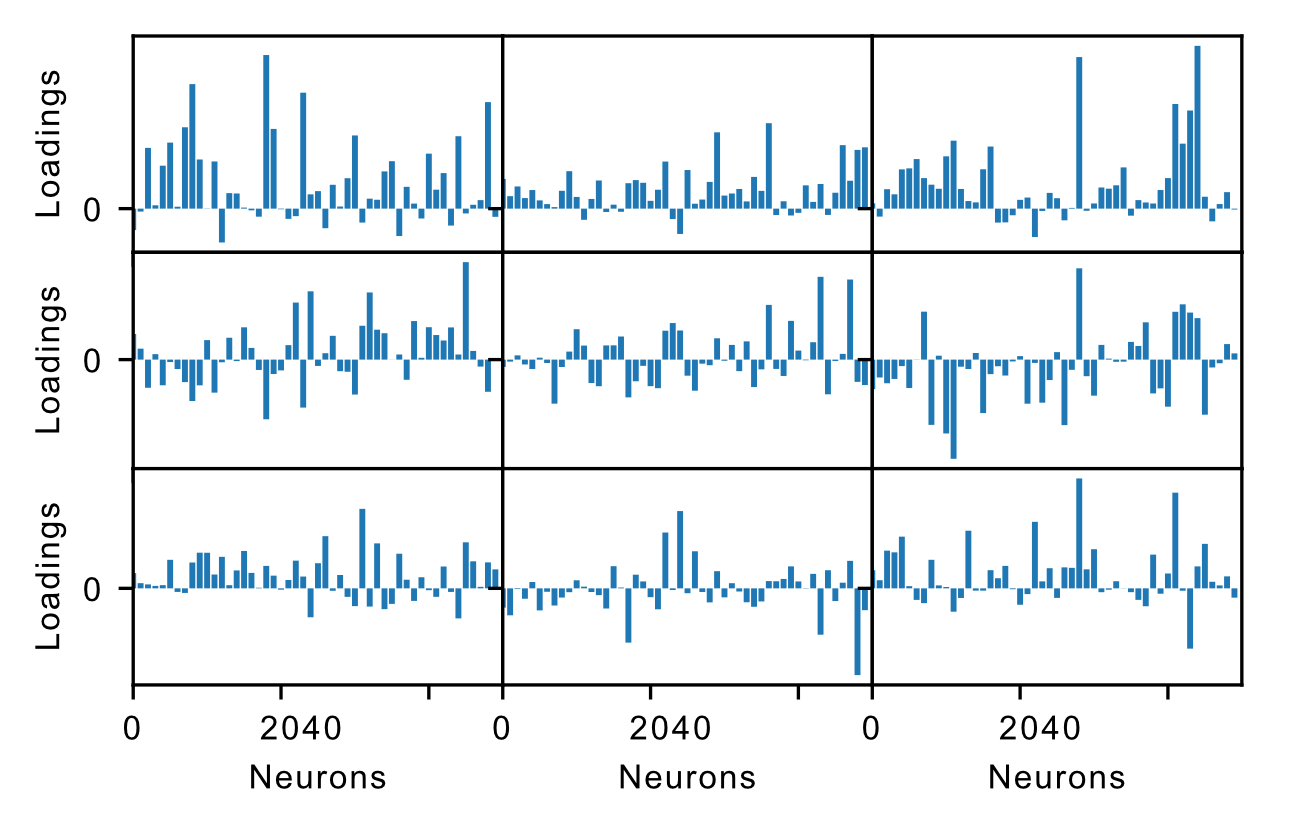

Supplement: Figure 7-1 — Loadings for the top three principal components. Each row represents a principal component, and each column is for a particular sample dataset, truncated to 50 neurons for clearer visualization. In all cases, the loadings are nonuniform with a mix of positive and negative contributions. Download Figure 7-1, TIF file. [file enu-eN-NWR-0287-21-s08.tif]
